# Molecular Epidemiology of Nontypeable *Haemophilus influenzae* Causing Community-Acquired Pneumonia in Adults

**DOI:** 10.1371/journal.pone.0082515

**Published:** 2013-12-13

**Authors:** Carmen Puig, Laura Calatayud, Sara Martí, Fe Tubau, Carolina Garcia-Vidal, Jordi Carratalà, Josefina Liñares, Carmen Ardanuy

**Affiliations:** 1 Department of Microbiology, Hospital Universitari de Bellvitge, Barcelona, Spain; 2 CIBER de Enfermedades Respiratorias (CIBERes), ISCIII, Madrid, Spain; 3 Epidemiology of Bacterial Infections Group, IDIBELL, Barcelona, Spain; 4 Department of Pathology and Experimental Therapeutics, Universitat de Barcelona, Barcelona, Spain; 5 Department of Infectious Diseases, Hospital Universitari de Bellvitge, Barcelona, Spain; 6 Spanish Network for Research on Infectious Diseases (REIPI), ISCIII, Madrid, Spain; Kliniken der Stadt Köln gGmbH, Germany

## Abstract

Nontypeable *Haemophilus influenzae* (NTHi) is an opportunistic pathogen which causes a variety of respiratory infections. The objectives of the study were to determine its antimicrobial susceptibility, to characterize the β-lactam resistance, and to establish a genetic characterization of NTHi isolates. Ninety-five NTHi isolates were analyzed by pulsed field gel electrophoresis (PFGE) and multi locus sequence typing (MLST). Antimicrobial susceptibility was determined by microdilution, and the *fts*I gene (encoding penicillin-binding protein 3, PBP3) was PCR amplified and sequenced. Thirty (31.6%) isolates were non-susceptible to ampicillin (MIC≥2 mg/L), with 10 of them producing β-lactamase type TEM-1 as a resistance mechanism. After *fts*I sequencing, 39 (41.1%) isolates showed amino acid substitutions in PBP3, with Asn526→ Lys being the most common (69.2%). Eighty-four patients were successfully treated with amoxicillin/clavulanic acid, ceftriaxone and levofloxacin. Eight patients died due either to aspiration or complication of their comorbidities. In conclusion, NTHi causing CAP in adults shows high genetic diversity and is associated with a high rate of reduced susceptibility to ampicillin due to alterations in PBP3. The analysis of treatment and outcomes demonstrated that NTHi strains with mutations in the *fts*I gene could be successfully treated with ceftriaxone or fluoroquinolones.

## Introduction

*Haemophilus influenzae* is a human-restricted pathogen which forms part of the normal nasopharyngeal microbiota [1]–[4]. This bacterial species is commonly divided into two different groups depending on the presence or absence of the polysaccharide capsule, with six serotypes (a–f) currently described in the encapsulated group. In children, serotype b (Hib) is responsible for most invasive diseases, although incidence has dramatically decreased since vaccine introduction [4]. Non-capsulated *H. influenzae*, also known as nontypeable *H. influenzae* (NTHi), colonizes asymptomatically the nasopharynx in healthy people, and is also a frequent cause of otitis media, sinusitis, conjunctivitis, community-acquired pneumonia (CAP) and exacerbations in chronic obstructive pulmonary disease (COPD) [1]–[4].

CAP is a common respiratory infection which frequently requires patient hospitalization. Current studies identify *H. influenzae* as either the second most common pathogen causing CAP, after *Streptococcus pneumoniae* [5], [6], or the third most common pathogen after *S. pneumoniae* and *Mycoplasma pneumoniae* [7]. In our geographical area, *H. influenzae* has been identified as the aetiological agent in 6–10% of CAP [8].

Aminopenicillin antibiotics have been used in the treatment of *H. influenzae* infections, and as a result, mechanisms of resistance against this group of antimicrobials have developed [9]–[11]. The most common mechanism of β-lactam resistance involves the production of a β-lactamase enzyme, usually TEM-1 type or, more rarely, ROB-1 type [12]. Alterations in penicillin-binding proteins (PBP3) have also been reported in different *H. influenzae* strains [13], [14]. This phenotype, also known as β-lactamase negative ampicillin resistance (BLNAR), is related to mutations in the *ftsI* gene (encoding the transpeptidase domain of PBP3) [15]. The frequency of resistance to other antimicrobials such as quinolones or azithromycin is, however, low [11], [16].

Epidemiological studies of individual patient groups are important for determining the level and mechanisms of antimicrobial resistance. In line with this goal, the present study had three main objectives: to determine the antimicrobial susceptibility of nontypeable *H. influenzae* strains isolated from patients with non-bacteremic CAP, to characterize the β-lactam resistance and to establish the clonal relatedness among these strains.

## Materials and Methods

### Ethics Statement

This work was approved by the ‘Comité Ètic d'Investigació Clínica del Hospital Universitari de Bellvitge’ and the written or oral informed consent was considered not necessary, because the source of bacterial isolates was anonymized and the study was retrospective.

### Hospital Setting and Bacterial Strains

This study was carried out at the Hospital de Bellvitge in Barcelona, a hospital for adults serving a population of ca. 600,000 people. A retrospective review of computerized medical charts was performed in all patients seen at the hospital during the study period in order to record those with CAP criteria. Pneumonia was considered when a new infiltrate on a chest radiograph plus one or more of the following symptoms were detected: fever or hypothermia, new cough, pleuritic chest pain, dyspnea or altered breath sounds on auscultation [8]. Overall mortality was defined as death within 30 days of pneumonia diagnosis. Patients were considered cured when clinical findings of pneumonia had disappeared and there was radiological improvement.

A total of 95 NTHi isolates were collected from sputum samples of 92 patients diagnosed with non-bacteremic CAP between 2000 and 2009.

Only *H.* influenzae isolates from good quality sputum samples (<10 squamous cells and >25 leukocytes per low-power field) and with a predominance of Gram negative coccobacilli forms were considered [17].

Isolates were identified by conventional methodology and preserved by cryopreservation. Additionally, all isolates were identified by mass spectrometry using a MALDI-Biotyper version 3.0 (Bruker), following the manufacturer's recommendations. Differentiation between *H. influenzae* and *H. haemolyticus* was performed by the detection of *fuc*K, *iga* and *lgt*C genes using a previously described methodology [18]. Isolates were identified as *H. influenzae* if they were positive for the three tested genes.

### Biotyping, Serotyping and Antimicrobial Susceptibility

Biotypes were determined using three biochemical reactions: urease, indol and ornithine decarboxylase [19]. Serotyping was achieved with the latex agglutination Phadebact® Haemophilus Test (Bactus AB, Huddinge, Sweden) and by PCR as stipulated by Falla et al. [20]. Antimicrobial susceptibility was determined by microdilution according to the criteria of the Clinical Laboratory Standards Institute (CLSI) [21], [22]. β-lactamase production was screened using the chromogenic cephalosporin method (nitrocefin disks, BD, Madrid, Spain).

### PCR and DNA Sequencing

Identification of β-lactamase type was performed by PCR on all the β-lactamase positive isolates using primers and conditions described previously [23]. For molecular characterization of PBP3, an internal region of the *ftsI* gene (796–1741 pb) was amplified by PCR and sequenced using previously described methodology [24].

### Genotype Definition for Ampicillin Resistance

According to previous descriptions [25], [26] and on the basis of β-lactamase production and changes in the *fts*I gene, *H. influenzae* isolates were classified into four genotypes: β-lactamase negative ampicillin susceptible (gBLNAS), strains without a detectable resistance mechanism; β-lactamase negative ampicillin resistant (gBLNAR), strains that did not produce a β-lactamase enzyme but which presented mutations in the transpeptidase domain of the *fts*I gene; β-lactamase positive ampicillin resistant (gBLPAR), strains producing β-lactamase but which did not present mutations in *fts*I; and β-lactamase positive amoxicillin/clavulanic acid resistant (gBLPACR), strains which presented both resistance mechanisms (β-lactamase production and mutations in the *ftsI* gene).

### Molecular Typing

#### Pulsed field gel electrophoresis (PFGE)

Strain relatedness was determined by PFGE with the restriction enzyme *SmaI* (New England BioLabs, Ipswich, MA, USA), as instructed by the manufacturer. Molecular typing was performed on bacterial suspensions of *H. influenzae* grown on chocolate agar plates, as described by Dabernat et al. [24] but with some modifications. Briefly, bacterial suspensions were prepared in PIV (10 mM Tris-HCl [pH 8], 1 M NaCl) and adjusted to the same final concentration. The bacterial suspension was mixed with an equal volume of melted 1.5% low-melting point agarose (Life Technologies, Madrid, Spain) in order to prepare DNA-agarose plugs with a volume of 20 µl each. These were incubated for 5 h at 37°C in 1 ml of ST buffer (6 mM Tris-HCl [pH 8]; 1 M NaCl; 0.1 M EDTA [pH 8]) containing 0.5% Brij-58, 100 µg/mL lysozyme and 50 µg/ml RNAse. The agarose plugs were transferred into ES buffer (1 M EDTA, 1% sarcosyl) with 1 mg/mL proteinase K (Sigma Aldrich, Madrid, Spain) and incubated over night at 50°C. Finally, the plugs were rinsed three times at room temperature with TE buffer (10 mM Tris-HCl [pH 8]; 1 mM EDTA [pH 8]).

The DNA-embedded plugs were digested with 5 U of *SmaI* for 18 h at 25°C. DNA fragments were then separated in a 1% agarose gel (Megabase, BioRad) with 0.5% TBE buffer (45 mM Tris-base, 45 mM boric acid, 1.0 mM EDTA pH 8.0) in a contour-clamped homogenous electric field system (CHEF DR III; BioRad). The gels were run for 19 h at 14°C, using a constant voltage of 6 V/cm with an angle of 120° and an increasing pulse time from 1 s to 30 s. A bacteriophage λ, low-range PFG marker (New England BioLabs, Ipswich, MA, USA) was used as a size standard.

PFGE band patterns were analyzed using the Fingerprinting II Software 3.0 (BioRad). The similarity of the PFGE banding patterns was estimated with the Dice coefficient, setting the optimization and tolerance at 1%. Isolates with ≥80% relatedness were considered highly genetically related [27].

#### Multilocus sequence type (MLST)

Clinical isolates were analyzed by MLST in order to identify strain relatedness [28]. Allele number and sequence types (ST) were assigned using the *H. influenzae* MLST website (http://haemophilus.mlst.net). The overall database was analyzed using e-BURST v3 in order to define groups available on the *H. influenzae* MLST website.

## Results

### Patient Characteristics and Antimicrobial Susceptibility

NTHi isolates were recovered from 95 episodes of CAP in 92 patients. Sixty-four patients (69.6%) were men and the mean age was 68.15 years (SD±14.39). Comorbid conditions were present in 97% of patients, with COPD being the most frequent underlying disease (28.3%), followed by chronic heart disease (18.5%), malignancy (15.2%), diabetes mellitus (13%) and chronic renal failure (4.3%). Finally, 59.7% of patients were either current (13%) or past (46.7%) smokers.

Table 1 summarizes the antibiotic susceptibility of the NTHi isolates. All of them were susceptible to ceftriaxone, cefotaxime and levofloxacin. By contrast, 10.5% of the isolates were resistant to ampicillin due to the expression of a TEM-1 β-lactamase, and 23.2% presented intermediate resistance. The rate of resistance to cotrimoxazole was high (32.6%), whereas the frequency of resistance to amoxicillin/clavulanic acid, cefuroxime, tetracycline, chloramphenicol and azithromycin was low (<4%).

**Table 1:** Minimal inhibitory concentrations (MIC) of 10 antimicrobials. MIC against 95 NTHi isolates using the microdilution method according to CLSI breakpoints.

Antimicrobials	MIC_50_	MIC_90_	Range	CLSI^a^	
	(mg/L)	(mg/L)		%I	%R
**Ampicillin**	0.5	2	≤0.25–≥16	23.2	10.5
**Amoxicillin/clavulanic acid** b	1	4	≤0.5–8	0	2.1
**Ceftriaxone**	<0.06	<0.06	≤0.06–0.12	0	0
**Cefotaxime**	<0.06	<0.06	≤0,06–0.12	0	0
**Cefuroxime**	2	4	≤0.5–≥8	3.1	1.1
**Tetracycline**	≤2	≤2	≤2–≥4	0	2.1
**Chloramphenicol**	≤2	≤2	≤2–8	0	1.1
**Azithromycin**	2	2	≤0.5–≥4	0	1.1
**Levofloxacin**	≤0.5	≤0.5	≤0.5–1	0	0
**Cotrimoxazole** c	≤0.5	>2	≤0.5–≥2	0	32.6

^a^ CLSI: Clinical and Laboratory Standards Institute. I: intermediate; R: resistant.

∶1.^b^ The ratio of amoxicillin/clavulanic acid was 2

∶19.^c^ The ratio of cotrimoxazole was 1

### Mutation Patterns in the *fts*I Gene

The sequence of *fts*I encoding the transpeptidase region of PBP3 was determined in all the isolates. Table 2 summarizes the amino acid changes observed, corresponding to 41.1% of the isolates. The most common amino acid substitution was Asn526→ Lys (27/39, 69.2%), followed by Arg517→ His (2/39, 5.1%). The patterns observed were classified into groups I and II according to the criteria of Dabernat et al. [24].

**Table 2:** Amino acid substitutions in the transpeptidase domain of PBP3 identified in 95 NTHi isolates.

Group^a^	Amino acid substitutions											MIC (mg/L)^b^		BL^c^	No isolates	Sequence Type (ST)
	Asp 350	Ala 368	Met 377	Met 391	Ala 545	Gly 490	Ala 502	Arg 517	Asn 526	Ala 530	Thr 532	AMP	AMC			
I								His				0.5–2	1–2	-	2	159 (n = 2)
IIa						Glu			Lys	Ser		2	4	-	1	14
									Lys	Ser		2	4	-	2	142, 414
									Lys			2	4	-	1	998
	Asn					Glu			Lys	Ser		1	1	-	1	201
IIb	Asn		Ile				Val		Lys			≥16	8	+	1	165
	Asn		Ile				Val		Lys			1–2	4	-	3	14, 142, 367
	Asn		Ile			Glu	Val		Lys			1–2	1–2	-	3	204, 556, 1177
IIc	Asn						Thr		Lys			≥16	8	+	1	1171
							Thr		Lys			2	2–4	-	8	1048, 993, 819 (n = 2), 1162, 996, 1000, 409
	Asn						Thr		Lys			1–2	1–4	-	6	556, 648, 1171, 999, 159 (n = 2)
Miscellaneous	Asn											≥16	4	+	1	997
		Thr										≤0.5	1	-	2	267, 1163
	Asn											≤0.5	≤0.5–1	-	2	388, 1143
				Ile								0.5	1	-	1	994
							Val					0.5	1	-	1	85
	Asn										Asn	0.5	1	-	1	425
					Val							≤0.25	≤0.5	-	2	991 (n = 2)
No changes												8–≥16	1–4	+	7	57, 142, 160, 270, 272, 836, 1172
												≤0.25–1	≤0.5–2	-	49	d

[24]; the miscellaneous group was classified according to the criteria of García-Cobos et al. [10] and the data from this study.^a^ The isolates were classified into groups I, IIa, IIb and IIc, according to the criteria of Dabernat et al.

≤1 mg/L; AMC Resistant: ≥8/4 mg/L; AMC susceptible: ≤4/2 mg/L;^b^ AMP Resistant: >4 mg/L; AMP Intermediate: 2 mg/L; AMP Susceptible

+: positive; -: negative);^c^ BL: Beta-lactamase production (

= 3), ST36, ST98, ST103, ST139 (n = 2), ST145 (n = 3), ST159 (n = 3), ST183, ST203 (n = 3), ST241 (n = 2), ST245, ST266, ST270, ST272, ST385, ST408, ST414 (n = 2), ST519 (n = 4), ST582, ST679, ST714, ST974, ST989, ST990, ST992, ST995, ST1174, ST1176, ST1178, ST1179, ST1180, ST1181, ST1182, ST1183 and ST1184.^d^ ST11 (n

Two isolates were classified as Group I and presented the Arg517→ His substitution alone. Group II included 27 isolates subdivided into three subgroups: i) 5 isolates belonged to the subgroup IIa (1 isolate with Asn526→ Lys, and the remaining 4 isolates with other mutations); ii) 7 isolates were classified as subgroup IIb, defined by Asn526→ Lys and Ala502→ Val substitutions (those isolates also presented the substitutions Asp350→ Asn and Met377→ Ile, and one of them also had a Gly490→ Glu); iii) the subgroup IIc, characterized by Asn526→ Lys and Ala502→ Thr substitutions, was the most common, with 15 isolates. No isolates were observed in subgroup IId or in groups III and III-like (previously described by García-Cobos et al. [10]).

Six patterns (10 isolates) were characterized and classified into the miscellaneous group: four of them (6 isolates) have already been described by García-Cobos et al. [10], while the remaining two were determined in this study and presented the Ala454→ Val and Asp350→ Asn/Thr532→ Asn substitutions.

Fourteen of 36 gBLNAR isolates (38.9%) presented ampicillin MIC within the susceptibility range (≤0.25–1 mg/L). All the isolates with MIC ≤0.25 or 0.5 of ampicillin belonged to the miscellaneous group, suggesting that these mutations were not involved in decreased β-lactam susceptibility.

### Phenotypic and Genotypic Characterization

Phenotypically, the most common biotype found was biotype II (39.0%) followed by biotypes III (35.7%), I (16.8%), V (3.2%), VI (3.2%) and IV (2.1%). As a result of positive detection of *lgt*C, *fuc*K and *iga* genes, all the isolates were identified as *H. influenzae*.

Molecular typing by PFGE revealed 47 different PFGE patterns. Twenty-six patterns were genotypically unique and 21 clusters contained between 2 and 15 related isolates (>80% similarity). Furthermore, molecular typing by MLST showed 67 different sequence types, with 28 of them (ST974, ST989 to ST1000, ST1143, ST1162, ST1163, ST1171, ST1172, ST1174, and ST1176 to ST1184) being described for the first time in the present study. The most frequent ST was ST159 (7 isolates). Analysis with e-BURST (including single and double locus variants) revealed 11 groups (≥2 isolates) and 29 singletons (only 1 isolate). Groups 1, 2 and 10 were the largest, with 9 isolates each (Table S1, Supplementary data).

**Figure 1:**
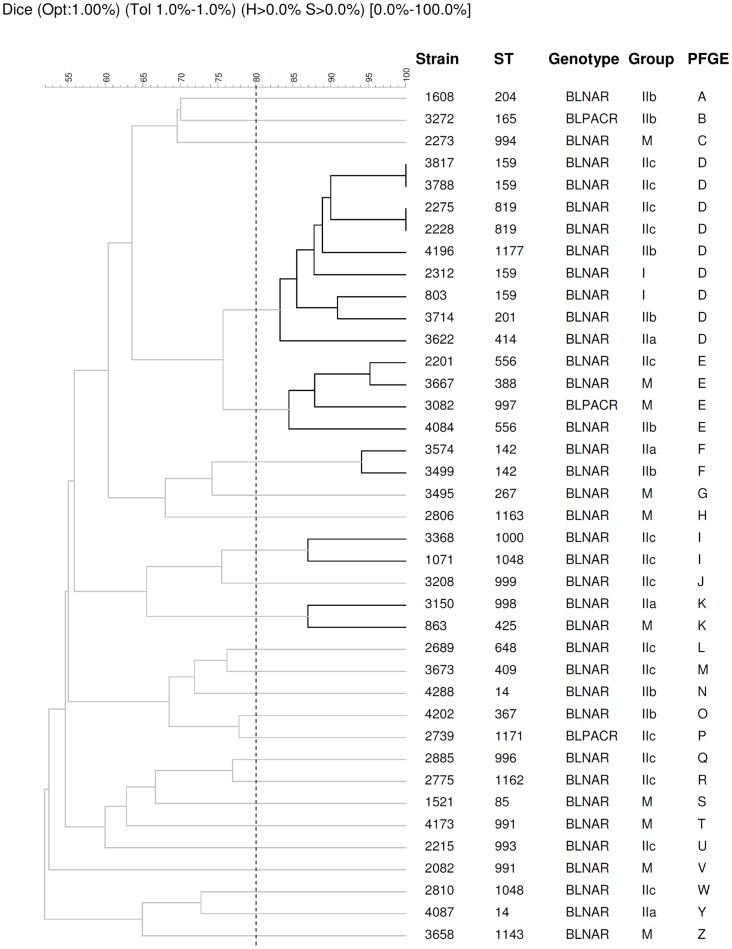
Tree diagram showing the genetic relatedness of 39 nontypeable *H. influenzae* isolates with mutations in the *fts*I gene (gBLNAR n = 36 and gBLPACR n = 3) obtained by PFGE according to Dice's similarity index. Dice coefficients are shown above the tree diagram. Isolates with ≥80% relatedness are considered highly genetically related.

The 39 isolates with mutations in the *fts*I gene were grouped into 25 independent PFGE clusters. Despite the fact that most patterns were unique, five clusters were identified with between two and nine genetically-related isolates (>80% similarity) (Figure 1). Cluster D grouped the majority of isolates with alterations in PBP3 (n = 9), with five different ST: ST159 (n = 4), ST819 (n = 2), ST201 (n = 1), ST414 (n = 1) and ST1177 (n = 1). These nine clonally-related isolates were collected from different patients throughout the study period. Six of these isolates were grouped in the same e-BURST group (ST159/ST819). The isolates in this cluster belonged to different amino acid substitution groups: IIc (n = 4), IIb (n = 2), I (n = 2) and IIa (n = I). Cluster E contained four isolates with three different ST: ST556 (n = 2), ST388 (n = 1) and ST997 (n = 1). Two of these isolates belonged to the miscellaneous group of amino acid substitutions, while the remaining two isolates belonged to subgroups IIb and IIc, respectively. The other three clusters (F, I and K) contained two isolates each. The isolates in cluster F had the same ST (ST142) and were classified into subgroups IIa and IIb. Cluster I comprised isolates with ST1000 and ST1048, which belonged to the same subgroup (IIc). Finally, cluster K was composed of isolates with ST425 and ST998, which were grouped into subgroup IIa and the miscellaneous amino acid substitution groups, respectively.

### Treatment and Patient Outcomes

Antibiotic therapy and clinical outcomes were analyzed for all patients included in this study. All patients were treated following the recommendations of the Infectious Disease Society of America and the guidelines of the American Thoracic Society [29].

Forty-one of 46 patients infected by gBLNAR, gBLPAR or gBLPACR isolates were successfully treated, mainly with amoxicillin/clavulanic acid, ceftriaxone and levofloxacin, or by using a combination of two of these antibiotics. The remaining five patients, infected by gBLNAR isolates, were treated with amoxicillin/clavulanic acid and ceftriaxone but died, due to aspiration, during the first 72 h of hospital admission (Table 3).

**Table 3:** Treatment and clinical outcomes for episodes of community-acquired pneumonia caused by NTHi.

Genotype^c^	Outcome	Treatment^a^				
		AMC	CRO	LEV	SXT	Combined therapy^b^
**gBLNAS**						
Cured	46	12	19	5	1	9
Died	3	2	1			
**gBLNAR**						
Cured	31	11	15	4		6
Died	5	3	2			
**gBLPAR**						
Cured	7	3	1			3
**gBLPACR**						
Cured	3	1	1	1		

^a^ AMC: amoxicillin/clavulanic acid; CRO: ceftriaxone; LEV: levofloxacin; SXT: cotrimoxazole.

β-lactam with fluoroquinolone or fluoroquinolone with another antibiotic.^b^ Combined therapy is

^c^ Genotypes are defined in the Materials and Methods section.

Forty-three of 46 patients infected by isolates with a genotype susceptible to aminopenicillins (gBLNAS) were successfully treated with ceftriaxone, amoxicillin/clavulanic acid and levofloxacin. The remaining three patients died by aspiration or due to complication of their severe underlying diseases (Table 3).

## Discussion

*H. influenzae* is a common cause of CAP in adults (6–10%) [8] and it is frequently associated with recurrent pneumonia in both children and adults [8], [30]. In this study, we analyzed the molecular epidemiology of NTHi causing non-bacteremic CAP in adult patients in the Barcelona area of Spain.

β-lactam antimicrobials are the first therapeutic option for treating CAP due to *H. influenzae* [29]. Resistance to ampicillin varies among European countries [31], [32]. The rate of reduced susceptibility to ampicillin found in this study was 33.7% (10.5% of isolates were resistant and 23.2% presented intermediate resistance), which is higher than the rate reported (16.2%) in a recent Spanish study by Perez-Trallero et al. [16]. A possible explanation for this high percentage of ampicillin non-susceptibility is that the majority of NTHi isolates were obtained from elderly patients who had received multiple antibiotic courses for their underlying diseases.

β-lactamase are the most common mechanism through which resistance to β-lactam antibiotics is acquired, although the frequency of their involvement fluctuates depending on the geographical area in question [33]–[35]. In our study, 10.5% of isolates presented TEM-1 β-lactamase production. This result is consistent with an overall downward trend that has been observed in Spain (from 25.7% in 1997 to 15.7% in 2007 [16]), as well as in other European countries and the USA [36]. However, different rates of β-lactam resistance due to alterations in PBP3 have been reported in several countries [15], [24], [36], [37]. In the present study, 41.1% of isolates had amino acid substitutions in the transpeptidase domain of PBP3. The percentage of BLNAR isolates detected in other European countries such as Germany (11.8%), France (0%), Portugal (9.6%) and the UK (1.5%) is lower than that found here [37]. The observed rate of gBLNAR could be due to the fact that most of our patients with CAP received multiple β-lactam antibiotic courses as treatment for their underlying diseases. Furthermore, the consumption of aminopenicillins in Catalonia increased from 46.1% in 1992 to 59.6% in 2007 [38], and this could also explain the frequency of gBLNAR observed in this study. In line with a previous report on Spanish isolates [10], the most frequent mutation found in the *fts*I gene was Asn526→ Lys, followed by Arg517→ His, and this allowed us to use the Dabernat et al. classification to group our isolates [24]. The presence of these mutations conferred a reduced susceptibility on ampicillin and amoxicillin/clavulanic acid (MIC between 1–4 mg/L) although those mutations alone were not enough to confer full resistance. In this set of NTHi, no isolates were found to belong to groups III or III-like (Met377→ Ile and Ser385→ Thr substitutions), which have been related to decreased cefotaxime and cefixime susceptibility [10].

Most of our patients infected with strains that were non-susceptible to ampicillin were successfully treated with amoxicillin/clavulanic acid, ceftriaxone or levofloxacin. In accordance with other studies [16], [31], amoxicillin/clavulanic acid, third-generation cephalosporins and quinolones showed excellent in vitro activity and are good therapeutic options for treating non-bacteremic CAP due to NTHi. However, since no gBLNAR isolates with ampicillin MIC ≥4 mg/L were found in our study, the clinical outcomes of patients infected by strains with high ampicillin MIC is unknown.

NTHi strains isolated from CAP episodes were found to be genetically diverse, this being consistent with other surveillance studies performed on respiratory or invasive NTHi isolates [39], [40]. Some studies carried out on BLNAR strains have demonstrated the high genotypic heterogeneity and lack of clonal spread in these strains [41], [42]. However, recent studies suggest a clonal dissemination of some BLNAR or BLPACR strains [10], [43], [44]. In our study, some small clusters of gBLNAR strains were found (Figure 1), but only one cluster, comprising two strains, presented the same *fts*I pattern, thereby suggesting a lack of clonal distribution in NTHi from CAP patients.

In conclusion, this study has established the genotypic characterization and antimicrobial resistance of NTHi causing non-bacteremic CAP in adult patients. The results illustrate the high genetic diversity among these strains, as well as the high rate of reduced susceptibility to ampicillin due to alterations in PBP3. Finally, the analysis of treatment and outcomes in this group of patients demonstrated that NTHi strains with mutations in the *fts*I gene (gBLNAR and gBLPACR) could be successfully treated with ceftriaxone or fluoroquinolones.

## Supporting Information

Table S1 **Groups based on e-BURST analysis with MLST data of 95 NTHi causing non-bacteremic CAP.**
(DOC)
