# Supplementary material for: Molecular Epidemiology of Nontypeable Haemophilus influenzae Causing Community-Acquired Pneumonia in Adults
Source: PLoS One. 2013 Dec 13;8(12):e82515. doi: 10.1371/journal.pone.0082515 (PMC3862678; doi:10.1371/journal.pone.0082515)
Supplement: Table S1 — Groups based on e-BURST analysis with MLST data of 95 NTHi causing non-bacteremic CAP. (DOC) [file pone.0082515.s001.doc]

| Group^a^ | Sequence type | Allelic profiles | | | | | | | No. isolates |
| --- | --- | --- | --- | --- | --- | --- | --- | --- | --- |
|  |  | *adk* | *atpG* | *frdB* | *fuck* | *mdh* | *pgi* | *recA* |  |
| 1 | ST14 | 5 | 1 | 1 | 1 | 1 | 2 | 5 | 2 |
|  | ST183 | 14 | 44 | 1 | 1 | 22 | 1 | 5 | 1 |
|  | ST267 | 1 | 5 | 1 | 1 | 1 | 83 | 5 | 1 |
|  | ST367 | 1 | 1 | 1 | 1 | 67 | 1 | 5 | 1 |
|  | ST408 | 1 | 1 | 1 | 1 | 1 | 21 | 5 | 1 |
|  | ST582 | 1 | 80 | 1 | 1 | 1 | 13 | 5 | 1 |
|  | ST974 | 1 | 1 | 1 | 1 | 73 | 42 | 5 | 1 |
|  | ST1171 | 1 | 107 | 1 | 1 | 22 | 1 | 5 | 1 |
| 2 | ST11 | 1 | 8 | 1 | 14 | 9 | 14 | 13 | 3 |
|  | ST103 | 1 | 1 | 1 | 14 | 9 | 14 | 13 | 1 |
|  | ST139 | 1 | 1 | 1 | 14 | 45 | 14 | 21 | 2 |
|  | ST145 | 1 | 8 | 1 | 14 | 22 | 14 | 13 | 3 |
| 3 | ST266 | 3 | 18 | 53 | 15 | 86 | 14 | 23 | 1 |
|  | ST993 | 3 | 18 | 53 | 15 | 86 | 14 | 3 | 1 |
|  | ST1182 | 3 | 18 | 53 | 15 | 231 | 14 | 23 | 1 |
| 4 | ST57 | 14 | 7 | 13 | 7 | 17 | 13 | 17 | 1 |
|  | ST98 | 14 | 7 | 13 | 15 | 17 | 13 | 1 | 1 |
| 5 | ST648 | 1 | 1 | 35 | 14 | 115 | 1 | 5 | 1 |
|  | ST679 | 1 | 1 | 10 | 14 | 186 | 1 | 5 | 1 |
| 6 | ST999 | 6 | 20 | 16 | 15 | 77 | 8 | 43 | 1 |
|  | ST1048 | 6 | 20 | 107 | 15 | 77 | 196 | 43 | 2 |
| 7 | ST388 | 60 | 51 | 16 | 48 | 15 | 1 | 31 | 1 |
|  | ST997 | 60 | 51 | 107 | 48 | 15 | 2 | 31 | 1 |
| 8 | ST1162 | 50 | 12 | 32 | 50 | 147 | 49 | 125 | 1 |
|  | ST1174 | 68 | 12 | 32 | 50 | 147 | 183 | 125 | 1 |
| 9 | ST245 | 1 | 24 | 18 | 18 | 27 | 1 | 5 | 1 |
|  | ST836 | 1 | 11 | 18 | 18 | 62 | 1 | 5 | 1 |
| 10 | ST159 | 33 | 8 | 16 | 16 | 17 | 2 | 29 | 7 |
|  | ST819 | 14 | 8 | 16 | 16 | 17 | 2 | 3 | 2 |
| 11 | ST409 | 1 | 1 | 1 | 14 | 15 | 111 | 5 | 1 |
|  | ST1163 | 50 | 11 | 1 | 14 | 15 | 1 | 5 | 1 |
|  | ST1176 | 160 | 1 | 1 | 14 | 15 | 1 | 5 | 1 |

^a^ The remaining 49 isolates were singleton: ST36, ST85, ST142 (n=3), ST160, ST165, ST201, ST303 (n=3), ST204, ST241 (n=2), ST270 (n=2), ST272 (n=2), ST385, ST414 (n=3), ST425, ST519 (n=4), ST556 (n=2), ST714, ST989, ST990, ST991 (n=2), ST992, ST994, ST995, ST996, ST998, ST1000, ST1143, ST1172, ST1177, ST1178, ST1179, ST1180, ST1181, ST1183, ST1184.
